# Subepidermal Calcified Nodule in a Child With Neurofibromatosis Type 1

**DOI:** 10.7759/cureus.23261

**Published:** 2022-03-17

**Authors:** Buket Bagci, Cansu Karakas, Murat Gokden

**Affiliations:** 1 Pathology, RWJBarnabas Health, Livingston, New Jersey, USA; 2 Pathology, University of Rochester Medical Center, Rochester, New York, USA; 3 Pathology and Neuropathology, University of Arkansas for Medical Sciences, Little Rock, Arkansas, USA

**Keywords:** calcinosis cutis, idiopathic calcinosis, calcium metabolism, neurofibromatosis, subepidermal calcified nodule

## Abstract

Calcinosis cutis (CC) is characterized by calcium deposition in the subcutaneous tissues. Subepidermal calcified nodule (SCN) is a variant of idiopathic calcinosis most commonly seen in the head and neck region of children and adolescents as a single, small, painless, yellow-white papule. A 13-year-old boy with a medical history of neurofibromatosis type 1 (NF1) presented with a firm 0.3 cm white papule in the lower eyelid. He also had neurofibromas of the left forearm and spinal cord, and a malignant peripheral nerve sheath tumor of the right forearm. The eyelid lesion showed hyperkeratotic epidermis, papillomatosis, and elongated rete ridges, with a radial arrangement at the periphery of the well-circumscribed lesion comprising many dystrophic calcifications, histiocytes, and foreign body giant cell reactions. To our knowledge, this is the first case of SCN reported in the context of NF1 or any other systemic disease in the English literature. Although a coincidence is likely, rare observations of the parathyroid gland and calcium metabolism disorders in association with NF1 may provide an explanation that requires further investigation.

## Introduction

Calcinosis cutis (CC) is a benign condition characterized by calcium deposition in the subcutaneous tissue, with metastatic, dystrophic, calciphylactic, iatrogenic, and idiopathic forms. Metastatic calcinosis is caused by hypercalcemia and hyperphosphatemia in otherwise normal tissue. Dystrophic calcinosis is caused by calcium deposition in tissues previously damaged by trauma or underlying disease, with normal serum calcium and phosphate levels. Iatrogenic CC develops following liver transplant and calcium chloride usage. Idiopathic calcinosis (IC) develops in a localized area with normal calcium and phosphate levels, and unclear etiology and pathogenesis. Subepidermal calcified nodule (SCN) is a form of IC most commonly affecting otherwise healthy children and adolescents with a mean age of 8.4 years, male:female ratio 2:1, and with no known systemic association [1,2]. It most commonly presents as a single, small, painless, yellow-white papule [3] developing in the head and neck region with the eyelid being the most common site. Rare locations such as knee, fingers, toes, soles, palms, oral mucosa, and penis have been documented [3-8]. Definitive diagnosis is by biopsy and microscopic examination, which shows calcium deposition in the dermis with or without chronic inflammation, and histiocytic reaction. Chronic inflammation and foreign body giant cell reaction are prominent in children and adolescents, and mild or absent in adults [9]. Here, we present a case of SCN in a 13-year-old boy with a history of neurofibromatosis (NF10, and a diagnosis of malignant nerve sheath tumor (MPNST). This case has been presented in part as a poster at the Annual Meeting of the American Society of Dermatopathology, Oct. 20-24, 2021.

## Case presentation

**Figure 1:**
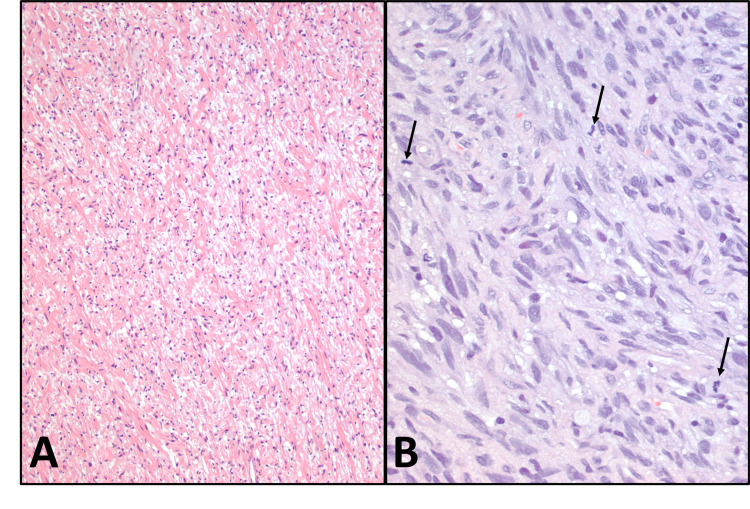
Histopathology of the peripheral nerve sheath tumors A: Neurofibroma from the left forearm. B: Malignant peripheral nerve sheath tumor from the right forearm with prominent cytologic atypia and increased mitotic activity (arrows). (Hematoxylin and eosin, original magnifications: A, 100x; B, 400x)

**Figure 2:**
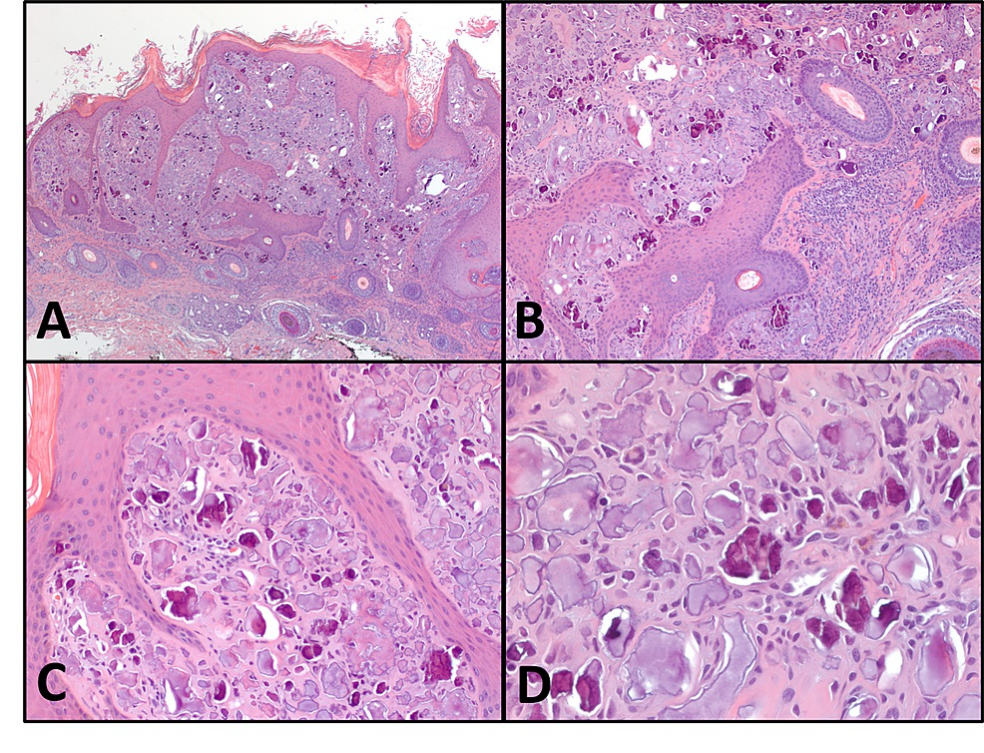
Histopathology of the eyelid lesion A: The lesion is situated in the superficial dermis, is well-circumscribed, and is associated with hyperkeratosis, elongation of the rete ridges, which are curved inward at the periphery. B: Many calcifications with chronic inflammatory infiltrate at the base of the lesion. C and D: Amorphous calcifications are admixed with histiocytes. (Hematoxylin and eosin; original magnifications: A, 20x; B and C, 100x; D, 400x)

A 13-year-old boy with autism, and NF1 diagnosed at three years of age, developed extensive neurofibromas over the years, involving soft tissues and intraspinal, prevertebral, and sacral nerve roots, lumbosacral plexus, and carotid sheath. A left forearm nodule was identified during his most recent clinical survey, which was excised to show a neurofibroma (Figure 1A). Approximately one month before this excision, a mass on his right distal forearm grew rapidly with heterogeneous cystic and necrotic changes seen on imaging. Microscopic examination of the right arm lesion showed an MPNST (Figure 1B) with atypical malignant cells, hypercellularity, increased mitotic activity, and necrosis. In addition to the already-known multiple café au lait spots, during his most recent visit, a 0.3 cm solitary papule 1.0 cm inferior to his right lateral canthus was identified and excised simultaneously with the left forearm neurofibroma, showing a raised nodular lesion in the superficial dermis, resulting in hyperkeratosis and elongation of rete ridges. Microscopic examination of the eyelid lesion (Figure 2) showed it to be situated in the superficial dermis, resulting in hyperkeratosis and elongation of rete ridges. It comprised numerous amorphous calcific deposits, intermingled with histiocytes. It was well-circumscribed with a flat base. Aggregates of mononuclear inflammatory infiltrate were present. Rare multinucleated histiocytes without foreign body giant cell reaction were identified. The diagnosis of SCN was made.

## Discussion

SCN is a rare form of IC. It was first described by Winer in 1952 as sweat gland hamartoma [10]. Woods and Kellaway in 1963 described it as SCN and suggested two mechanisms for its formation [11]. The first mechanism is that calcified granules within the stroma create a larger calcified mass through calcification and degeneration of stroma. Alternatively, the large calcified lesion occurs first and its subsequent reabsorption leaves the calcified granules that form the calcified nodule with associated tissue changes. In 1980, Tezuka explained the mechanism as deposition of calcium and phosphate from the degranulated mast cells that form SCN [12]. Increased levels of gamma carboxyglutamic acid (G1a) have been found to be elevated in soft tissue calcifications and, with its calcium and phospholipid binding properties, it has been suggested as a potential cause for ectopic soft tissue calcifications [13]. The definitive etiology and pathogenesis of SCN are still unknown.

This entity most commonly affects the younger population. In a previous study, Evans et al. identified 21 cases of SCN age varied between 1-17 years old (mean 8.4 years old) with male dominance (14M/7F), 14 of which occurred in the head and neck region [4]. Shields et al. reported a mean age of 12 years in six cases of periocular SCN [14]. Khine et al. demonstrated the male predominance (67% male vs 33% female) and most of the patients (89%) were younger than 21 years of age [3]. In terms of age, gender, and location; our case was consistent with the previously reported findings. The clinical diagnosis of SCN can be challenging due to the rarity of these lesions. It usually presents as a solitary nodule or, rarely, as multiple yellow-white, firm nodules [8]. It can also present as a skin-colored or erythematous nodule [15]. When it presents as a single nodule, it can clinically mimic epidermal inclusion cyst, calcifying pilomatrixoma, hemangioma, or seborrheic keratosis [8,9,14]. When it presents as multiple nodules, the differential diagnosis includes verrucous papilloma, xanthomas, molluscum contagiosum, and milia. In our case, the clinical impression was neurofibroma due to prior history of multiple neurofibromas and the presence of a firm skin-colored papule.

Histopathologically, SCN is characterized by amorphous calcium deposits within the dermis. AlWadani et al. demonstrated that histopathologic examination of these lesions in children showed lymphoplasmacytic inflammation and foreign body giant cell reaction surrounding the calcium deposition, especially in long-standing lesions, whereas in adults, fibrous tissue was surrounding the calcium deposition without the presence of inflammation or foreign body giant cell reaction [9]. Additionally, in children, multiple small deposits were present, whereas, in adults, a single large mass forming calcium deposit was seen [9]. A previous study by Evans et al. demonstrated in 21 cases that, lesions in younger patients showed a more warty appearance with the calcium predominantly deposited in large amorphous clumps, whereas in older adults, calcium was deposited in small globular spherules [4]. Our case presented with many small deposits of calcium and a chronic inflammation predominantly consisting of lymphocytes and plasma cells. No foreign body giant cell reaction was identified. Although its histology is quite pathognomonic, as with our case, histologic differential diagnosis of SCN may include other entities that may have some calcification component or other depositions mimicking calcifications, such as eruptive xanthoma, gout, and oxalosis. These entities have their typical clinical and pathologic features and should not pose a significant problem in the differential diagnosis of SCN.

In the literature, no known association of SCN with specific systemic diseases exists; however, some variants of CC can present in association with metabolic conditions that cause calcium imbalance, such as renal disease, and rheumatologic diseases such as systemic sclerosis [4,16,17]. The association of SCN with gout and hypertension is suggested in the literature, but a definitive relationship couldn’t be concluded [9]. In our case, the patient was diagnosed with NF1 10 years before calcified nodules appeared and continuously developed new neurofibromas throughout his body, as well as an MPNST. Additionally, the neurofibroma on his right arm developed approximately at the same time as the SCN. The development of recent MPNST and a concurrent neurofibroma at the time of diagnosis of SCN may be concerning for a possible association of SCN with neurofibromatosis. Abnormalities of calcium metabolism and parathyroid gland adenomas have been reported in association with NF1 [18-20]. On the other hand, these abnormalities would result in metastatic calcification in multiple sites as a result of hypercalcemia and hyperphosphatemia, both of which have been within normal limits in our patient. Although not specifically investigated, there is no indication of parathyroid hyperplasia or neoplasia in multiple radiologic surveys performed for NF1. As such, it is not possible to prove any causal relationship between SCN and NF1, and a mere coincidence is likely in this case. Nonetheless, we were not able to identify a report of SCN in the setting of NF1 in the English literature.

Indications for treatment of SCN are impairment of function due to location, the presence of pain, and, most commonly, cosmetic reasons. Treatment modalities for SCN include but are not limited to excisional biopsies, intralesional corticosteroid injections, and carbon dioxide laser application. Most of the time excisional biopsy is the treatment of choice for diagnostic purposes as clinical diagnosis can be challenging. Although recurrence is not common, it can occur after incomplete excision [8].

## Conclusions

SCN is a rare idiopathic nonneoplastic entity, with no definitive association with a systemic disease, including neurofibromatosis. Although disorders of calcium metabolism have been observed in the setting of NF1, no definitive connection with SCN nor explanation of its formation in this setting is apparent from the literature. To our knowledge, this is the first report of an SCN with such co-occurrence. Careful observations and biopsy of dermatologic lesions in future cases should increase our experience and provide additional support to any connection between these two conditions.
